# Resolving Viral-Induced Secondary Bacterial Infection in COPD: A Concise Review

**DOI:** 10.3389/fimmu.2018.02345

**Published:** 2018-10-16

**Authors:** Hao Wang, Desiree Anthony, Stavros Selemidis, Ross Vlahos, Steven Bozinovski

**Affiliations:** School of Health and Biomedical Sciences, RMIT University, Melbourne, VIC, Australia

**Keywords:** COPD-chronic obstructive pulmonary disease, exacerbation, influenza (flu), secondary infection, co-infection, resolvin D1 (RvD1), serum amyloid A (SAA), pneumococcus (*Streptococcus pneumoniae*)

## Abstract

Chronic obstructive pulmonary disease (COPD) is a leading cause of disability and death world-wide, where chronic inflammation accelerates lung function decline. Pathological inflammation is worsened by chronic bacterial lung infections and susceptibility to recurrent acute exacerbations of COPD (AECOPD), typically caused by viral and/or bacterial respiratory pathogens. Despite ongoing efforts to reduce AECOPD rates with inhaled corticosteroids, COPD patients remain at heightened risk of developing serious lung infections/AECOPD, frequently leading to hospitalization and infection-dependent delirium. Here, we review emerging mechanisms into why COPD patients are susceptible to chronic bacterial infections and highlight dysregulated inflammation and production of reactive oxygen species (ROS) as central causes. This underlying chronic infection leaves COPD patients particularly vulnerable to acute viral infections, which further destabilize host immunity to bacteria. The pathogeneses of bacterial and viral exacerbations are significant as clinical symptoms are more severe and there is a marked increase in neutrophilic inflammation and tissue damage. AECOPD triggered by a bacterial and viral co-infection increases circulating levels of the systemic inflammatory marker, serum amyloid A (SAA). SAA is a functional agonist for formyl peptide receptor 2 (FPR2/ALX), where it promotes chemotaxis and survival of neutrophils. Excessive levels of SAA can antagonize the protective actions of FPR2/ALX that involve engagement of specialized pro-resolving mediators, such as resolvin-D1. We propose that the anti-microbial and anti-inflammatory actions of specialized pro-resolving mediators, such as resolvin-D1 should be harnessed for the treatment of AECOPD that are complicated by the co-pathogenesis of viruses and bacteria.

## COPD patients are highly susceptible to respiratory infections

Chronic obstructive pulmonary disease (COPD) is an umbrella term encompassing multiple lung pathologies (including emphysema, chronic bronchitis and bronchiolitis) that manifest into persistent and poorly reversible airflow limitation. It is a major chronic disease that is predicted to become the third leading cause of death world-wide by 2030 (1) and has a huge economic burden costing $50 billion annually in the USA alone (2). COPD is characterized as a chronic inflammatory condition due to the persistent accumulation of innate and adaptive immune cells in the airways along with increased systemic inflammation (3). This inflammatory process is driven, in part by inhalation of highly noxious particles in cigarette smoke and biomass fuels that overwhelm protective detoxifying and anti-oxidant pathways. Inflammation is maintained through the continual recruitment and activation of leukocytes, which perpetuate a state of oxidative stress and local tissue damage. Neutrophilic inflammation is particularly prominent in COPD where neutrophils degranulate with increasing severity of COPD, resulting in uncontrolled release of proteolytic enzymes (neutrophil elastase and matrix metalloproteinase 9-MMP9) that further damage the lungs (4).

An important paradox in COPD is that despite the accumulation of leukocytes in the airways with increasing disease severity, there is still a major failure to adequately control and eradicate respiratory pathogens. As a consequence, the lower airways become persistently infected, where up to 50% of COPD patients are chronically colonized with potentially pathogenic bacteria including *Haemophilus influenzae, Streptococcus pneumoniae* and *Moraxella catarrhalis* (5, 6). Another major consequence of susceptibility to infection is that people with underlying severe disease frequently develop acute exacerbations of COPD (AECOPD). AECOPD are defined as “a sustained worsening of the patient's condition, from the stable state and beyond normal day-to-day variations, that is acute in onset and necessitates a change in regular medication in a patient with underlying COPD” (7). AECOPD are predominately caused by acquisition of a new respiratory pathogen (8), which accelerate pathological remodeling leading to a more rapid decline in lung function (9). Severe exacerbations account for much of the health related expenditure for COPD (10) and the “frequent exacerbator” phenotype has been defined, where patients that fall into this category are at much greater risk of hospitalization and death (11).

Anti-inflammatory agents including inhaled corticosteroids in combination with bronchodilators are frequently used to treat COPD, however they have failed to reduce the rate of severe AECOPD requiring hospitalization (12). Inhaled corticosteroids, delivered alone or in combination with bronchodilators are also associated with a small but significant increased risk of serious adverse pneumonia events (13). *S. pneumoniae* is the major bacterial cause of community acquired pneumonia (CAP); accounting for up to 40–50% of bacteriologically examined cases. COPD is a common underlying chronic comorbid condition in CAP (14) and the severity of CAP is worse in COPD patients based on severity index and respiratory failure (15). Without radiological assessment, it is difficult to differentiate CAP from AECOPD, however CAP is generally associated with more severe illness and pulmonary consolidation. Polyvalent vaccination may also be less effective in COPD patients as the elevated relative risk for developing pneumococcal pneumonia in COPD (8-fold compared to controls) is only reduced by 50% with vaccination (16). With over 90 serotypes, the genetic diversity of *S. pneumoniae* means that strategies in addition to vaccination are needed to control this pathogen, particularly in people with chronic comorbid conditions, such as COPD.

## Dysfunctional oxidant pathways disrupt clearance of respiratory pathogens in COPD

The persistence of bacterial infection in the lower airways of COPD patients is associated with impaired phagocytic function, where clearance of both *H. influenzae* and *S. pneumoniae* by alveolar macrophages was shown to be defective relative to control macrophages (17, 18). Oxidative stress is thought to drive impaired phagocytosis as activation of nuclear erythroid–related factor 2 (Nrf2) restores phagocytosis of bacteria by alveolar macrophages from patients with COPD (19). Nrf2 is a key transcription factor that regulates expression of a suite of cytoprotective and antioxidant enzymes. As COPD progresses in severity, inflammatory cells accumulate and become an important endogenous source of reactive oxygen species (ROS) independent of smoking status. Increased ROS production by activated leukocytes will also promote the peroxidation of polyunsaturated fatty acids and generation of reactive carbonyl species, such as 4-hydroxynonenol (4HNE) and malondialdehyde (MDA); both of which are increased in COPD (20). Levels of auto-antibodies against carbonyl-modified proteins correlate with severity of COPD (21). Importantly, reactive carbonyls can impair macrophage-mediated phagocytosis of bacteria by directly causing cytoskeletal instability and carbonyl modification of pseudopodia (22–24). Also, macrophages interact with carbonyl-modified extracellular matrix proteins and this interaction suppresses their phagocytic functions (25).

Immunity to common respiratory viruses is also disrupted in COPD patients. Around 30–50% of AECOPDs are associated with respiratory viruses including rhinovirus, respiratory syncytial virus (RSV) and influenza (26, 27). Experimental rhinovirus infection in subjects with COPD results in elevated systemic and airway inflammation (28). In addition, increased rhinovirus load was detected in COPD subjects compared to controls, which was associated with reduced interferon production (28). Using the same human experimental model, rhinovirus infection was also shown to markedly increase markers of oxidative and nitrosative stress (29). We have recently shown that single stranded RNA viruses (including influenza A virus, rhinovirus, RSV) and DNA viruses promote the production of ROS through activation of endosomal NADPH oxidase 2 (NOX2) (30). The internalization of viruses specifically triggered production of hydrogen peroxide within endosomes, where NOX2-dependent production of ROS suppressed antiviral signaling networks via modification of Toll-like receptor-7 (30). This process acts as a critical physiological brake to prevent an over exuberant anti-viral or humoral response that can contribute to autoimmune disease. Conversely, excessive ROS production in COPD has the potential to compromise essential anti-viral immune responses (such as type-1 interferon) during viral-induced exacerbations, although this has yet to be proven. We have however, targeted excessive ROS generation with the glutathione peroxidase (Gpx) mimetic and NOX2 oxidase inhibitor, ebselen and apocynin in an experimental AECOPD model (31). Here, we exposed mice to cigarette smoke prior to acute influenza A virus infection, which resulted in a greater viral lung burden (31). Furthermore, treatment with ebselen effectively reduced viral lung titres and lung inflammation, thus identifying the Gpx pathway as a novel therapeutic target for the treatment of viral-induced exacerbations (31).

## Viral and bacterial co-infections increase the severity of AECOPD

AECOPD associated with respiratory viruses including rhinoviruses have been shown to be frequently followed by secondary bacterial infections in COPD (32). A subsequent study revealed that acute rhinovirus infection during AECOPD promoted a significant rise in pathogenic bacteria, such as *H. Influenzae* from the pre-existing lung microbiota, which persisted for over 40 days (33). Hence, the classic secondary bacterial infection setting whereby an acute viral event leads to a secondary bacterial lung infection may not necessarily represent the natural course of respiratory co-infections in COPD. This is because many moderate/severe COPD patients are already chronically infected with multiple pathogenic bacteria before they encounter a viral pathogen. Acute flares or COPD exacerbations associated with the presence of bacterial and viral pathogens occur frequently, constituting around a quarter of infective exacerbations (8, 34, 35), and this rate may increase with the development of more sensitive assays to detect respiratory pathogens. Clinically, AECOPD associated with the presence of a bacterial and viral pathogen are significant as they result in more severe events involving greater lung function impairment and longer hospitalization (8). Virus infection in the presence of chronic bacterial infection is also an important determinant of hospital readmission following the initial exacerbation (36). AECOPD associated with respiratory co-infections result in increased bacterial lung loads during the acute phase of the exacerbation (37). The viral-mediated outgrowth of bacteria is not restricted to rhinovirus and *H. Infuenzae* interactions as rhinovirus can also facilitate acquisition and transmission of *S. pneumoniae* (38). Influenza A readily triggers pneumococcal dispersion from nasopharyngeal biofilms deeper into the lower airways (39). In addition, rhinovirus has been shown to impair antibacterial responses to the TLR4 ligand, LPS and reduce uptake of *E. coli* bioparticles (40).

At a cellular level, severe exacerbations drive an airway inflammatory response involving increased neutrophilic inflammation (36). The amplified neutrophilic response during severe AECOPD is thought to directly compromise host immunity to bacterial respiratory pathogens. We have previously shown that the degranulation of neutrophil-derived proteinases, such as neutrophil elastase and MMP9 increase with the severity of COPD irrespective of elevated corticosteroid therapy (4). Increased levels of neutrophil elastase are also detected during AECOPD associated with the presence of a viral and bacterial pathogen (32). In this cohort, the levels of antimicrobial peptides were markedly reduced in exacerbations where viral and bacterial pathogens were detected, and they suggest that uncontrolled degranulation of neutrophil elastase actively degrades anti-microbial peptides in the lung, leading to outgrowth of bacteria (32). Neutrophils can also increase mucin production by activating epidermal growth factor receptor (EGFR) signaling (41), which involves protease-dependent release of membrane bound EGFR ligands, such as TGF-α (42, 43). In addition, neutrophil-derived myeloperoxidase can also catalyze the generation of hypochlorous acid that cross-links mucus to form hydrogels (44). Since mucus hypersecretion is significantly associated with an excess decline in lung function during AECOPD and an increased risk of subsequent hospitalization (45), we propose that an excessive neutrophilic response is directly driving mucus-mediated obstruction during AECOPD.

## Targeting bacterial super-infections with specialized pro-resolving mediators

Hence, a therapeutic strategy that dampens the exuberant neutrophilic response during AECOPD may not only be beneficial in reducing the risk of developing a secondary bacterial infection, but will also alleviate symptomatic lung function decline due to excessive mucus production and plugging. To support our claim, we have actively screened for inflammatory biomarkers that are altered during AECOPD and identified serum amyloid A (SAA) as an acute phase reactant that is markedly increased during severe episodes (34). Furthermore, we demonstrated that the circulating levels of SAA were significantly higher in AECOPD associated with the presence of a bacterial and viral co-infection (34). SAA serves multiple innate host defense roles during acute infection including opsonisation of gram-negative bacteria to facilitate more efficient phagocytosis (46). It can also promote the recruitment of leukocytes to the site of infection as it is a functional agonist for the Formyl peptide receptor 2 (FPR2/ALX), which stimulates chemotaxis (47). In COPD, we found a positive association between SAA expression and neutrophilic inflammation in lung tissue biopsies derived from COPD patients (48). We demonstrated that SAA-induced recruitment of neutrophils into the airways is dependent on Interleukin 17A (IL-17A) signaling (48, 49). SAA can also prolong neutrophil survival by suppressing the apoptotic machinery involving activation of the ERK and PI3K/Akt signaling pathways (50). Hence, production of SAA is normally protective and self-limiting with a sharp decline during the resolution phase of infection, however the persistent elevation during AECOPD triggered by co-infections may lead to the excessive recruitment of neutrophils.

The persistence of SAA expression during AECOPD will alter FPR2/ALX signaling in innate and mucosal cells that express this receptor. FPR2/ALX is a member of the G-protein coupled receptor (GPCR) superfamily characterized by seven putative trans-membrane domains that displays diverse ligand affinities, interacting with over 30 different ligands (51). By binding to distinct ligands that are temporally expressed, this receptor is a master regulator of acute inflammation and resolution of inflammation. Hence, there is a class switch from pro-inflammatory ligands (such as SAA and the leukotriene LTB_4_) to pro-resolving mediators during the course of infection and acute inflammation (52). Lipoxin A_4_ (LXA_4_) is a specialized pro-resolving mediator that directly interacts with FPR2/ALX to initiate resolution pathways. This eicosanoid opposes leukocyte migration and activation by suppressing transendothelial (53) and transepithelial (54) migration and neutrophil degranulation (55). Furthermore, we have shown that LXA_4_ can potently antagonize the recruitment of neutrophils into the lungs in response to SAA stimulation (56). LXA_4_ also promotes tissue repair by stimulating basal cell proliferation required for wound healing following mucosal injury (57). In addition, LXA_4_ stimulates more efficient macrophage-mediated efferocytosis of apoptotic neutrophils, which is an essential component to the resolution of inflammation (58, 59).

FPR2/ALX also interacts with an alternate specialized pro-resolving mediator called resolvin D1 (RvD1), which belong to the D series resolvins derived from the omega-3 fatty acid, docosahexaenoic acid (50). Like LXA_4_, RvD1 and its more stable aspirin-triggered derivative (AT-RvD1) display potent anti-inflammatory actions in a number of disease models including cigarette smoke exposure and acute lung injury (60, 61). This eicosanoid is also a promising therapeutic target in bacterial pneumonia because it can stimulate macrophage phagocytosis of pathogenic bacteria (*E. coli, P. aeruginosa*, and *H. Influenzae)* and enhance neutrophil efferocytosis to prevent collateral lung injury (62, 63). More recently, we have evaluated the efficacy of AT-RvD1 in a co-infection model where mice were initially inoculated with *S. pneumoniae* and subsequently infected with influenza A virus. This model represents the AECOPD setting where the lower airways are chronically infected with pathogenic bacteria prior to encountering a respiratory virus. Similar to classic bacterial super-infection models, acute viral infection resulted in a marked increase in pneumococcal lung load (64). We also observed a significant increase in neutrophil and monocyte infiltration into the lungs of co-infected mice and the lung pathology was consistent with severe pneumonia. Levels of SAA were strikingly increased in the serum and lungs of co-infected mice relative to mice infected with either pneumococcus or influenza A virus alone (64). Our approach to reducing excessive inflammation in co-infected mice was to therapeutically deliver exogenous AT-RvD1 during the acute phase of infection, where FPR2/ALX is the only characterized receptor for AT-RvD1 in mice. AT-RvD1 reduced pneumococcal lung load and potently reduced the degree of pneumonia or alveolitis, which was associated with a marked reduction in neutrophil and monocyte lung infiltration in co-infected mice (64). The reduction in neutrophilic inflammation was accompanied by a reduction in neutrophil elastase activity in the lungs. The AT-RvD1 mediated suppression of neutrophil elastase activity was concurrently associated with restoration of anti-microbial activity in the bronchoalveolar lavage (BAL) fluid of co-infected mice (64). AT-RvD1 is also known to stimulate the production of the anti-bacterial peptide lipocalin 2, thereby enhancing anti-microbial activity (62).

In addition to reducing the degree of localized pneumonia, specialized pro-resolving mediators, such as AT-RvD1 may also treat the central consequences of co-infection or bacterial super-infection. In the classic bacterial super-infection setting, *S. pneumoniae* can enter and proliferate in the brain, with the ensuing inflammatory response causing bacterial meningitis in severe cases (65). Whilst meningitis rarely occurs, severe respiratory infections will prolong sickness behavior including fever, malaise and fatigue. The onset of sickness behavior is mediated in part, by inflammatory cytokines, such as IL-1β produced at the primary site of infection, which then act centrally to stimulate neuroinflammation (66). Brain inflammation can also produce acute cognitive impairments, such as delirium, which is common in COPD patients (67, 68) and this is worsened during acute exacerbations (69). The circulating inflammatory mediators that impair cognitive function during AECOPD are not well-characterized. However, circulating SAA can readily cross the intact blood-brain barrier (70) and excessive production of SAA in a transgenic model resulted in greater deposition in the brain and an increase in brain inflammation (71). Consistent with this study, we found that in our co-infection model associated with a marked increase in circulating SAA, there was an increase in SAA immuno-reactivity, increased numbers of “activated” amoeboid-shaped microglia and inflammatory cytokine expression in the brain (72). Furthermore, under *in vitro* conditions SAA proved to be a potent stimulus for primary mouse microglia activation, and this response was markedly suppressed by the anti-inflammatory actions of AT-RvD1 (72).

## Concluding remarks

**Figure 1:**
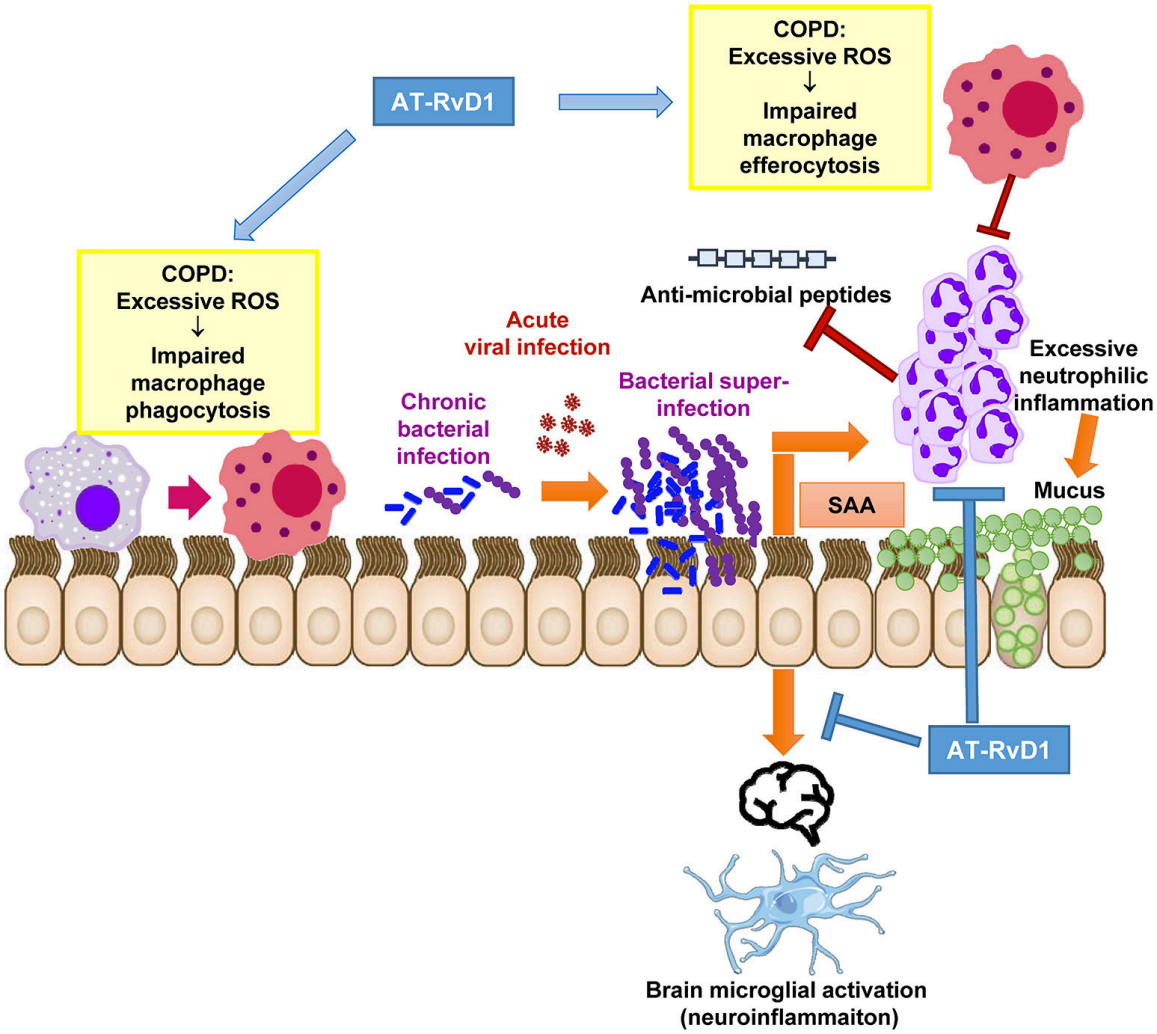
Proposed therapeutic actions of AT-RvD1 during acute exacerbations in chronically infected COPD patients. Excessive inflammation and ROS are a characteristic feature of the COPD lung microenvironment. Excessive ROS directly impairs macrophage function preventing efficient phagocytosis of potentially pathogenic bacteria and efferocytosis (removal of dying neutrophils). This has deleterious effects on mucosal immunity and permits the establishment of chronic bacterial infection of the lower airways. Upon exposure to a newly acquired viral infection, which is a common trigger for AECOPD, the virus permits the further outgrowth of bacteria causing a bacterial super-infection in the lungs. SAA is significantly increased during co-infections and stimulates neutrophilic inflammation via FPR2/ALX-dependent mechanisms. Excessive neutrophilic inflammation can drive mucus hypersecretion and degrade anti-microbial peptides in the airways. We propose that the alternative FPR2/ALX agonist, AT-RvD1 can therapeutically intervene at critical pathological pathways that lead to bacterial super-infections. AT-RvD1 facilitates the resolution of inflammation during co-infections by improving the phagocytic clearance of bacteria and efferocytosis of apoptotic neutrophils in the lungs. It also potently suppresses neutrophil migration, thereby limiting tissue damage, mucus secretion and anti-microbial peptide degradation caused by ongoing inflammation. AT-RvD1 may also reduce neuroinflammation consequent to serious lung co-infections as brain microglia are activated by SAA in manner that is suppressed by AT-RvD1.

The likelihood of developing a co-infection or secondary bacterial infection is increased in COPD largely due to the ROS-dependent suppression of macrophage phagocytic function. Suppressed macrophage phagocytosis contributes to the establishment of chronic bacterial infection of the lower airways. Consequently, acute infection with a newly acquired respiratory virus, such as influenza A can promote uncontrolled bacterial outgrowth in the lung. This is observed clinically, as the detection of a respiratory virus and bacteria during AECOPD occurs frequently and is associated with an increase in exacerbation severity and neutrophilic inflammation. We have shown that the exuberant inflammatory response during co-infections are associated with elevated levels of SAA, which is an agonist for FPR2/ALX that promotes neutrophil migration and survival. Specialized pro-resolving mediators, such as lipoxins and D-series resolvins can counteract the pro-inflammatory actions of SAA. We provide compelling pre-clinical data to demonstrate that AT-RvD1 is a very effective therapeutic in the co-infection setting, where it potently suppressed leukocyte tissue accumulation in the lungs and concurrently improved pneumococcal clearance. Furthermore, co-infections markedly increase brain inflammation and AT-RvD1 can suppress the release of inflammatory cytokines from activated microglia. In summary, specialized pro-resolving mediators have great potential in the co-infection setting as they not only reduce local tissue inflammation and improve bacterial clearance at the site of infection, but they can also dampen central microglial inflammation that prolongs sickness behavior and cognitive dysfunction (Figure 1).

## Author contributions

SB performed the literature review. HW, DA, and SB designed the figures. SB, SS, HW, DA, and RV organized, wrote and edited the manuscript.

### Conflict of interest statement

The authors declare that the research was conducted in the absence of any commercial or financial relationships that could be construed as a potential conflict of interest.
